# Andrographolide promotes proliferative and osteogenic potentials of human placenta-derived mesenchymal stem cells through the activation of Wnt/β-catenin signaling

**DOI:** 10.1186/s13287-021-02312-x

**Published:** 2021-04-14

**Authors:** Naruphong Phunikom, Nittaya Boonmuen, Pakpoom Kheolamai, Kanoknetr Suksen, Sirikul Manochantr, Chairat Tantrawatpan, Duangrat Tantikanlayaporn

**Affiliations:** 1 Division of Cell Biology, Faculty of Medicine, Thammasat University, Pathumthani, 12120 Thailand; 2 Center of Excellence in Stem Cell Research, Thammasat University, Pathumthani, 12120 Thailand; 3 Department of Physiology, Faculty of Science, Mahidol University, Bangkok, 10400 Thailand

**Keywords:** Andrographolide, Placenta-derived mesenchymal stem cells, Osteogenic differentiation, Regenerative medicine

## Abstract

**Introduction:**

The in vitro expansion and differentiation of mesenchymal stem cells derived from bone marrow (BM-hMSCs) are considered as potential therapeutic tools for clinical applications in bone tissue engineering and regenerative medicine. However, invasive sampling and reduction in number and proliferative capacity with age are the major limitations of BM-hMSCs. Recently, human placenta-derived MSCs (PL-hMSCs) obtained by a non-invasive procedure have attracted much interest. Attempts to increase the potential of PL-hMSCs would be an important paradigm in regenerative medicine. Herein, we examined the proliferative and osteogenic effect of andrographolide (AP) on PL-hMSCs.

**Methods:**

Mesenchymal stem cells were isolated from full-term normal human placentas and were characterized before using. Cell cytotoxicity and proliferative effect of AP were examined by MTT and BrdU assay, respectively. The non-toxicity concentrations of AP were further assessed for osteogenic effect determined by alkaline phosphatase (ALP) expression and activity, alizarin red staining, and osteoblast-specific gene expressions. Screening of genes involved in osteogenic differentiation-related pathways modulated by AP was explored by a NanoString nCounter analysis.

**Results:**

PL-hMSCs generated in this study met the MSC criteria set by the International Society of Cellular Therapy. The non-cytotoxic concentrations of AP on PL-hMSCs are up to 10 μM. The compound increased PL-hMSC proliferation concomitant with increases in Wnt/β-catenin level and activity. It also enhanced osteogenic differentiation in association with osteoblast-specific mRNA expression. Further, AP promoted bone formation and increased bone structural protein level, osteocalcin, in osteoblastic cells. Gene screening analysis showed the upregulation of genes related to Wnt/β-catenin, TGFβ/BMP, SMAD, and FGF signaling pathways.

**Conclusion:**

We demonstrated, for the first time, the potential role of AP in promoting proliferation, osteogenic differentiation, and osteoblast bone formation of PL-hMSCs. This study suggests that AP may be an effective novel agent for the improvement of PL-hMSCs and stem cell-based therapy for bone regeneration.

**Supplementary Information:**

The online version contains supplementary material available at 10.1186/s13287-021-02312-x.

## Introduction

Bone degenerative diseases as well as osteoporosis have a substantial impact on the quality of life and are major public health challenges [1, 2]. Bone marrow-derived mesenchymal stem cells (BM-hMSCs) have a direct role in the maintenance of bone balance and act as a source of progenitors for osteoblasts, which are responsible for bone formation and as regulators for osteoclastogenesis [3]. A decline in the number of BM-hMSCs is one of the important factors contributing to age-related bone loss, such as post-menopausal osteoporosis, because of a reduction in proliferative capacity accompanied by a decrease in osteogenic differentiation potential [4, 5]. BM-hMSCs are, therefore, considered as potential therapeutic tools for clinical applications in bone tissue engineering and regenerative medicine due to their beneficial characteristics including high self-renewal and differentiation abilities as well as low immunogenicity properties [6–9]. Nevertheless, major restrictions for clinical uses are the invasive procedure for cell harvesting, limited amounts of the bone marrow, and reduced proliferation capacity with age [2]. Therefore, alternative sources containing cells with higher proliferative potency, capability of differentiation, and lower risk of viral contamination are being considered. Currently, other sources of MSCs can be readily isolated from many different tissues including the adipose, cartilage, skin, muscle, and gestational tissues such as the umbilical cord and placenta [10]. The human placenta is a very attractive source of MSCs for several advantages such as easy accessibility, noninvasive procedures, a high-yielding source of stem cells, and minimal ethical controversies [11, 12].

PL-hMSCs have been reported to have the characteristics matching human MSC defined in 2006 by the Cell Committee of the International Society for Cellular Therapy (ISCT) [11]. In short, MSCs must adhere to plastic, express specific MSC cell surface markers, and have the potential of differentiating into chondrocytes, osteocytes, and adipocytes [13]. In addition, isolated PL-hMSCs represent a more homogeneous and primitive population that maintains MSC stemness compared to other tissues [14, 15]. Several studies have indeed revealed that PL-hMSCs have more proliferative capacity, longer life span, and immunomodulatory potential compared to other sources [16, 17]. Also, PL-hMSCs have a limited capacity to grow in culture, and therefore have lower chance of undergoing oncogenic transformation after transplantation, making them a safer product to be used in regenerative medicine compared with the pluripotent stem cells [18]. Currently. PL-hMSCs have been studied in a variety of disorders such as neurological diseases, cancer, cardiac diseases, and bone and cartilage diseases [19–23]. However, the increasing demand for PL-hMSCs for clinical trials makes high quality and large numbers of the cells mandatory. Therefore, it is necessary to explore safe and effective agents to improve the potential of PL-hMSCs for stem cell-based therapy in bone diseases.

Currently, several natural small compounds are being explored as new osteogenic inducers in stem cells. Andrographolide (AP), which is a diterpenoid lactone derived from *Andrographis paniculata* Nees, is one of the popular compounds that exerts several pharmacological activities including anti-inflammation, anti-oxidation, and immunomodulation [24–26]. It has been extensively used for treatments of inflammatory diseases such as fever, inflammation, diarrhea, and osteoarthritis [27, 28]. AP has been examined in various experimental studies on humans and animals indicating that it is safe and has less serious side effects [24, 29]. Recently, we have reported the osteogenic effect of AP in mouse pre-osteoblast cell lines and the protective effect on bone loss in estrogen-deficient rats [30]. However, the effects of AP on PL-hMSCs are still unknown. Therefore, we aimed to investigate the potential role of AP in in vitro PL-hMSC cell proliferation and in the ability to differentiate into osteoblasts.

## Materials and methods

### Isolation and culture of mesenchymal stem cells derived from placenta

Full-term normal human placentas were collected from pregnant women after normal deliveries at the Thammasat Chalermprakiat Hospital. Written informed consents were obtained from the mothers. This study was approved by the Human Ethics Committee of Thammasat University No. 1 (Faculty of Medicine; No.071/2017), which was in accordance with the Declaration of Helsinki, the Belmont Report, and ICH-GCP. Placenta-derived cells were prepared as follows. The placental tissue was chopped into small pieces and incubated with 0.25% (w/v) trypsin-EDTA (GIBCO™, Invitrogen Corporation, USA) for 30 min at 37 °C. The chopped pieces were washed twice with PBS and cultured in DMEM+ 10% (v/v) FBS (GIBCO™, Invitrogen Corporation, USA) in a 25 cm^2^ culture flask (Corning, USA). Cells were then cultured at 37 °C. To remove non-adherent cells, the media were changed every 3 days. The adherent cells were further cultured until colonies of fibroblast-like cells were obtained. For expansion, the cells were sub-cultured using 0.25% trypsin-EDTA. The morphology of PL-hMSCs was observed and photographed under an inverted microscope (Nikon Eclipse Ts2R, Japan). Culture cells were observed continuously to procure developing colonies of fibroblast–like cells.

### Immunophenotypical characterization of PL-hMSCs by flow cytometry

The phenotype of PL-hMSCs was evaluated by flow cytometry (FACScalibur™, Becton Dickinson, USA) and CellQuest® software (Becton Dickinson, USA). Native third to fifth passages of PL-hMSCs were trypsinized using 0.25% trypsin-EDTA and suspended in PBS. Cells were incubated with fluorochrome-labeled mouse anti-human monoclonal antibodies: anti-CD45-FITC (Bio Legend, USA), anti-CD34-PE (Biolegend, USA), anti-CD90- PE (Bio Legend, USA), anti-CD73-PE (Bio Legend, USA), and anti-CD105-PE (BD Bioscience, USA) for 30 min at 4 °C in the dark. After incubating with the antibodies, cell pellets were washed twice with PBS and fixed with 1% (w/v) paraformaldehyde in PBS.

### Adipogenic and osteogenic differentiation ability of PL-hMSCs

PL-hMSCs (3rd–6th passages) were used to evaluate their adipogenic and osteogenic differentiation potentials. Cells were cultured in 6-well plates with growth medium (10% FBS + DMEM) at a density of 5 × 10^3^ cells/cm^2^. For adipogenic differentiation, after cells reached 70–80% confluence, the medium was replaced with adipogenic medium containing DMEM (high glucose) supplemented with 10% FBS, 0.5 mM isobutylmethylxanthine, 100 nM dexamethasone, 1 μg/ml insulin solution, and 100 μM indomethacin (Sigma-Aldrich, USA). Adipogenic media were changed every 3 days, and the generation of lipid droplets was revealed by Oil Red O staining (Sigma-Aldrich, USA) after treatment for 28 days. For osteogenic differentiation, after cells reached ~ 90% confluence, the medium was changed to the osteogenic differentiation medium consisting of DMEM (low glucose) plus 10% FBS, 100 nM dexamethasone (Sigma-Aldrich, USA), 10 mM β-glycerophosphate (Sigma-Aldrich, USA), and 50 μg/ml ascorbic acid (Sigma-Aldrich, USA). Cells were cultured in the osteogenic differentiation medium for 21 days and the medium were changed every 3 days. Differentiated cells were analyzed by alizarin red staining and observed under an inverted microscope (Nikon Eclipse Ts2R, Japan).

### Cell viability and proliferation assays

Cell viability was detected by the MTT method [31]. Briefly, PL-hMSCs were seeded at 1 × 10^3^ cells/well in 96-well plates (Costa, Corning, USA) with growth medium. Twenty-four hours later, cells were treated with AP (Sigma-Aldrich, USA) dissolved in 0.05% methanol at the final concentrations of 0.1–50 μM and incubated for 24–120 h. Cells in the same volume of untreated culture media containing 0.05% methanol were used as control. After incubating for the indicated times, cells were incubated with MTT (0.5 mg/ml) for 4 h. The formazan precipitate was dissolved in 150 μL DMSO, and the absorbance was detected at 570 nm. The IC_50_ values were calculated according to the dose-dependent curves. All tests were repeated in at least three independent experiments. Cell viability (%) was calculated against untreated control cells by following formula.

$$ \%\mathrm{cell}\ \mathrm{viability}=\frac{\left(\mathrm{OD}\ \mathrm{in}\ \mathrm{test}\ \mathrm{well}-\mathrm{OD}\ \mathrm{in}\ \mathrm{blank}\ \mathrm{well}\right)}{\left(\mathrm{OD}\ \mathrm{in}\ \mathrm{untreated}\ \mathrm{control}\ \mathrm{well}-\mathrm{OD}\ \mathrm{in}\ \mathrm{blank}\ \mathrm{well}\right)}\;\mathrm{X}\ 100 $$

Cell proliferation was measured using a bromodeoxyuridine (BrdU) cell proliferation assay kit (Sigma-Aldrich; Merck KGaA) according to the manufacturer’s instruction. The measurements were performed in triplicates.

### ALP staining and enzyme activity assay

PL-hMSCs were cultured in 6-well plates at a density of 5 × 10^3^/cm^2^ in osteogenic condition for 14 days. For alkaline phosphatase (ALP) staining assay, the cultured cells were washed with PBS and fixed with 4% paraformaldehyde for 5 min at 4 °C. Then, 5-bromo-4-chloro-3-indolylphosphate/nitro blue tetrazolium liquid substrate (BCIP/NBT; Sigma-Aldrich, USA) was added and incubated for 30 min at room temperature. The reactions were stopped by rinsing with deionized water and observed under a light microscope (Nikon TS100, Japan). ALP activity assays were performed by using SensoLyte® pNPP Alkaline Phosphatase Assay Kit (Anaspec, Inc., USA). Briefly, the lysed cells were mixed with p-nitrophenyl phosphate (pNPP) substrate solution at room temperature for 30 min. After enzymatic reaction was stopped, absorbance values were measured at 405 nm. ALP activity was calculated from a p-nitrophenol (pNP) standard curve and normalized with total protein concentration measured by using bicinchoninic acid (BCA) assay kit (Sigma-Aldrich, USA).

### Alizarin Red S staining

To detect calcium deposition or mineralized matrix formation in osteoblasts, cells were washed with PBS then fixed with 4% paraformaldehyde. The fixed cells were stained with 2% Alizarin Red S for 20 min at room temperature, then washed twice with deionized water, and observed under a microscope. For the quantification of the desorbed calcium ions, ARS-stained cells were incubated with 10% cetylpyridinium chloride solution (pH 7.0) for 30 min. The extracted stain was transferred to a 96-well plate, and the absorbance was read at 570 nm. The level of ARS stain extracted from the osteogenic control cell cultures was considered as 100%.

### The effect of AP on Wnt/β-catenin transcriptional activity

The effect of AP on Wnt/β-catenin transcriptional activity was determined by luciferase reporter gene assay. HEK293T cells were seeded in 96-well culture plates at a density of 2.5 × 10^3^ cells/well and incubated for overnight. According to the instructions of the manufacturer, cells were then transiently transfected using Lipofectamine 2000 with TOPflash or FOPflash (0.5 mg), Renilla luciferase reporter plasmid (0.5 mg), and β-catenin-FLAG plasmid (0.1 mg) for 24 h. Cells were then treated with different concentrations of AP for 24 h, then cells were harvested with lysis buffer. The luciferase activities were measured with the dual-luciferase reporter assay system (Promega, Madison, WI, USA) using a luminometer (TECAN spark 10 M, TECAN, Mannedorf, Switzerland). The luciferase activity was normalized to Renilla luciferase activity as an internal control and expressed as the fold change compared with the cells transfected with the pcDNA3.1 empty vector.

### Total RNA isolation and quantitative PCR of genes

Total RNA was isolated using Trizol reagent the instructions of the manufacturer (Trizol, Invitrogen). RNA was quantified by A260 and reverse transcription used 500 ng of total RNA using an iScript select cDNA synthesis kit (Bio-Rad Laboratories Inc). Gene expressions were analyzed by quantitative RT-PCR using iTaq Universal SYBR Green Supermix (Bio-Rad Laboratories Inc.) and performed in ABI step one plus and analysis software (Applied Biosystems, Foster City, CA, USA). Relative mRNA quantity was calculated by the comparative cycle threshold (CT) method (ΔCT) using glyceraldehyde-3-phosphate dehydrogenase (GAPDH) as control. Sequences of primers for RT-PCR are shown in Table 1.

**Table 1:** Sequences of primers for quantitative RT-PCR analyses

Gene	Sequence (5′ to 3′)	Length of product (bp)
*Runx2*	F-5′-CCTCGGAGAGGTACCAGATG-3′ R-5′-TTCCCGAGGTCCATCTACTG-3′	247
*OSX*	F-5′-GCCAGAAGCTGTGAAACCTC-3′ R-5′-GCTGCAAGCTCTCCATAACC-3′	161
*ALP*	F-5′-CCTTGCTCACTCACTCACTCC-3′ R-5′-TTTTTTTTGCCGTTCCAAAC-3′	182
*Osteocalcin*	F-5′-GTGCAGAGTCCAGCAAAGGT-3′ R-5′-TCAGCCAACTCGTCACAGTC-3′	152
*Col1a1*	F-5′-AGGGCCAAGACGAAGACATCCC-3′ R-5′-TGTCGCAGACGCAGATCCG-3′	108
*OPG*	F-5′-AACGCCAACACAGCTCACAAGAAC-3′ R-5′-TGCTCGAAGGTGAGGTTAGCATGT-3′	160
*RANKL*	F-5′-ATCGTTGGATCACAGCACATC-3′ R-5′-AGACTCACTTTATGGGAACCAGA-3′	152
*C-myc*	F-5′-ATGGCCCATTACAAAGCCG-3′ R-5′-TTTCTGGAGTAGCAGCTCCTAA-3′	175
*AXIN2*	F-5′-CCTGGCTCCAGAAGATCACA-3′ R-5′-AGCATCCTCCGGTATGGAAT-3′	120
*Survivin*	F-5′-TGAGAACGAGCCAGACTTG-3′ R-5′-TGTTCCTCTATGGGGTCGTCA-3′	87
*Cyclin D1*	F-5′-GATCAAGTGTGACCCGGACTG-3′ R-5′-CCTTGGGGTCCATGTTCTGC-3′	101
*GAPDH*	F-5′-GAGTCAACGGATTTGGTCGT-3′ R-5′-TTGATTTTGGAGGGATCTCG-3′	238

### Western blot analysis

The expressions of active-β-catenin and β-catenin proteins were investigated by Western blot analysis. After seeding PL-hMSCs in 6-well plates and incubating with AP at the concentration 1, 2.5, 5, and 10 μM for 24 h, cells were harvested for protein assay. Briefly, cells were lysed with RIPA lysis buffer with a protease and phosphatase inhibitor cocktail (Roche diagnostic GmbH, Boehringer, Mannheim, Germany) and were incubated for 20 min on ice. Lysates were centrifuged to remove cell debris. Protein concentrations were determined using by bicinchoninic acid dye binding (BCA assay, Pierce, Rockford, IL). For Western blot, each sample contained 20 μg of protein. Lysates were diluted with 6X sample buffer, separated by 10% SDS-polyacrylamide gel electrophoresis, and transferred onto PVDF membranes. Membranes were blocked with 5% nonfat dried milk and then incubated with primary antibodies overnight at 4 °C. Antibodies were used as following: anti-active-β-catenin (clone 8E7) (Millipore, Darmstadt, Germany), anti-β-catenin (Santa Cruz Biotechnology, Inc., Dallas, TX, USA), and anti-β-actin (Sigma-Aldrich, St. Louis, MO, USA). Membranes were then incubated with the horseradish peroxidase-conjugated secondary antibodies for 1 h and visualized by enhanced chemiluminescence (Pierce ECL, Thermo-Fischer, Rockford, IL). The expression of β-actin was used as the control.

### Osteocalcin concentration determination

PL-hMSCs were cultured and prepared for the ARS assay as described above. Cell supernatants were collected for the determinations. The levels of secreted osteocalcin were determined by an enzyme-linked immunosorbent assay in accordance with the manufacturer’s recommended protocols (Elabscience Biotechnology Co., Wuhan, Hubei, China). Three duplicated wells were set for each group, and the concentrations were calculated based on standard curve.

### Nanostring® nCounter assay

Modulation of gene expression by AP on PL-hMSCs was determined by NanoString® nCounter Technology (NanoString Technologies, Seattle, WA, USA) using nCounter® PanCancer Panel. Multiplex gene expression analysis with 770 genes to assess proliferative and osteogenic genes of PL-hMSCs was performed. Hybridization of samples was performed, and the obtained products were run according to the manufacturer’s instructions. Data were collected on the nCounter Sprint profiler and further evaluated by nSolver software, v4.0 analysis. Transcript copies were normalized using the geometric mean of 29 housekeeping genes for reference and normalization. Fifty threshold count value was the background thresholding parameter; the fold changes of gene expression were calculated comparing treated samples with untreated controls. Raw *P* values from the differential expression analyses were used to assess gene expression data. All heat maps and data cluster sets were produced using the nCounter Analysis and Advanced Analysis packages in nSolver4.0 (NanoString Technologies, Seattle, WA, USA).

### Statistical analysis

The data are presented as mean ± standard error of the mean (SEM). Statistical comparisons were performed using the one-way ANOVA followed by a Newman-Keuls multiple comparison tests. *P* value of less than 0.05 was considered to be statistically significant.

## Results

### Characteristics of PL-hMSC phenotypes

**Fig. 1:**
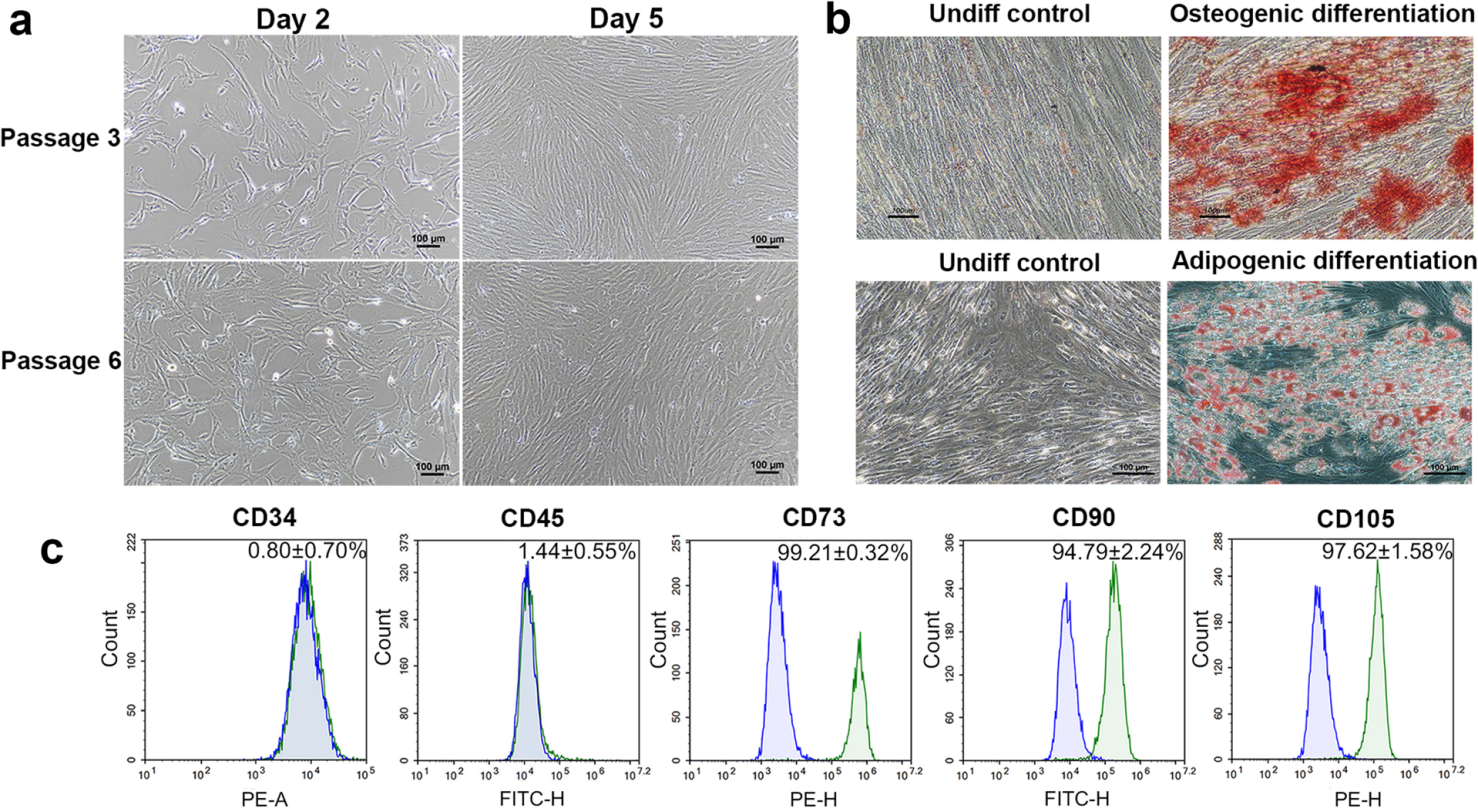
Identification of human placenta-derived mesenchymal stem cells (PL-hMSCs). **a** Appearance and growth of PL-hMSCs at 3rd and 6th passages on days 2 and 5, respectively. **b** Differentiation potentials of PL-hMSCs; representative images of alizarin red staining cultured in osteogenic induction medium after 21 days compared to control condition (left) and oil red O staining cultured in adipogenic induction medium after 28 days compared to control medium (right); scale bar, 100 μm. **c** The expressions of typical cell surface markers of MSC in PL-hMSCs. Detection of typical cell surface markers of MSC in PL-hMSCs (green) and in isotype controls (blue) by flow cytometry. PL-hMSCs expressed CD73, CD90, and CD105 but not CD34 and CD45

PL-hMSCs were isolated and expanded in primary cultures and passaged for 3–5 times. We found that the cells exhibited typical fibroblastic-like morphology, which attached, spread, and displayed spindle-shaped morphology on plastic surface of the tissue culture flasks (Fig. 1a). Further, the cells expressed typical surface markers according to the standard definition of MSCs including CD73, CD90, and CD105, but did not express hematopoietic markers including CD34 and CD45 (Fig. 1c). The results indicate that the cells used in this study are indeed PL-hMSCs.

Moreover, the differentiation potentials of PL-hMSCs were also examined. Under certain conditions, MSCs are characterized as multipotent cells which can differentiate into different cells. In our study, after PL-hMSCs were cultured under adipogenic condition for 4 weeks, cells were changed from spindle shaped morphology to large round shaped cells showing the accumulation of lipid droplets in their cytoplasm (Fig. 1b). In contrast, lipid droplets were not found in the control cultured cells. Calcium deposits are an indication of successful in vitro bone formation and can be specifically visualized using alizarin red staining. In this study, the calcium depositions were detected in osteogenic treated cells after 3 weeks (Fig. 1b), but they were not found in control cells. These results show that PL-hMSCs had the potential to differentiate into adipocyte and osteoblast cells upon proper stimulations. Therefore, PL-hMSCs generated in our study met the MSC criteria set by the International Society of Cellular Therapy and can be used for further investigations.

### Effect of AP on cell viability and proliferation

**Fig. 2:**
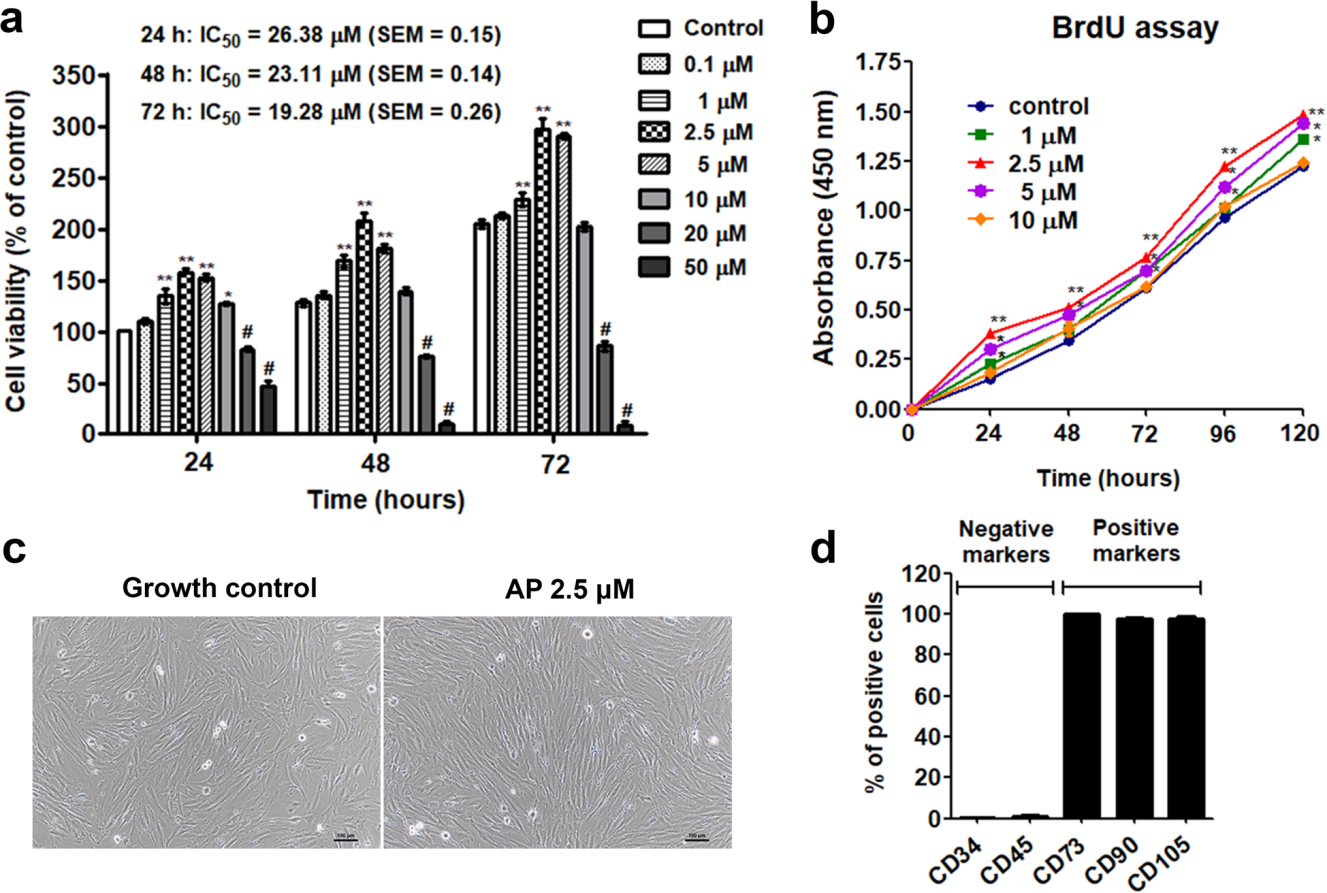
Effect of AP on cell viability and proliferation of PL-hMSCs. **a** Cell viability of PL-hMSCs in growth medium with various concentrations of AP (0.1–50 μM) at 24, 48, and 72 h. **b** Cell proliferation of PL-hMSC in growth medium with various concentrations of AP (1, 2.5, 5, 10 μM) at 24, 48, 72, 96, and 120 h. Data are presented as the means of independent experiments (*n* = 4). *P < 0.05, ***P < 0.01, and ***P < 0.001 significant increases compared to the control group at the same time point, #*P* < 0.05, significant decreases compared to the control group at the same time point. **c** Cell morphology of PL-hMSCs after treatment with 2.5 μM AP for 5 days. **d** The % of cell surface markers expressions in PL-hMSCs after treatment with 2.5 μM AP for 5 days performed by flow cytometry (positive for CD73, CD90, and CD105; negative for CD34 and CD45). Data are expressed as mean ± SEM; *n* = 3

Figure 2a demonstrates cell viability of PL-hMSCs treated with AP in growth medium for 24–72 h compared to control group. Interestingly, viability of the cells treated with 1–5 μM of AP were significantly increased compared to the control group (*P* < 0.05 and *P* < 0.01) but were decreased at higher concentrations (10–50 μM AP) suggesting that AP exhibits cell toxicity at the concentration higher than 10 μM. The half maximal inhibitory concentration (IC_50_ values) of AP at 24, 48, and 72 h were 26.38 ± 0.15, 23.11 ± 0.14, and 19.23 ± 0.26, respectively. To avoid any cytotoxic effects, the maximum dose of AP used in further experiments was 10 μM. Of note, the enhancements of cell viability were associated with increases in cell proliferation as confirmed by BrdU assay (Fig. 2b). We found that proliferations of PL-hMSCs cultured in growth medium containing 1, 2.5, and 5 μM of AP were significantly increased after treatment for 24, 48, 72, 96, and 120 h compared to the control group (*P* < 0.05 and *P* < 0.01). These results indicate that AP has a proliferative effect on PL-hMSCs. Moreover, we also examined the MSC characteristics of PL-hMSC at day 5 after treatment with 2.5 μM of AP. Interestingly, the cells still showed MSC characteristics in both morphology and phenotypic surface markers (Fig. 2c, d). The results suggest that AP has the ability to increase in vitro MSC expansion without alteration of the MSC stemness.

### AP increased the level and activity of β-catenin protein

**Fig. 3:**
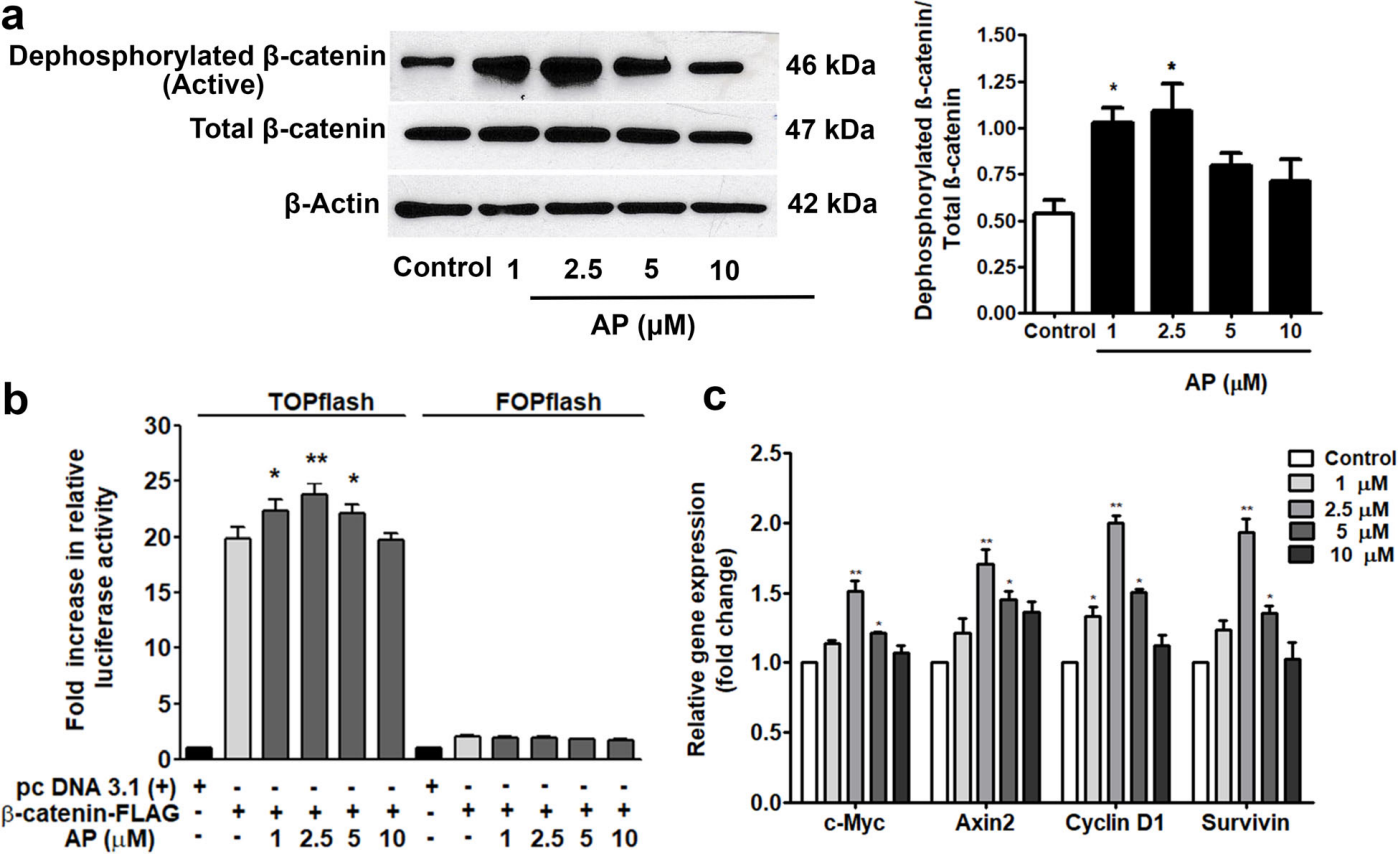
Effect of AP on Wnt/β-catenin during proliferation of PL-hMSCs. **a** Western blot assay showing the expressions of dephosphorylated β-catenin (active), total β-catenin, and β-actin after treatment with AP for 24 h (left panel) and the band intensity of dephosphorylated β-catenin (active)/total β-catenin ratio (normalized with β-actin; right panel). **b** Luciferase activity assay of β-catenin after treatment with AP for 24 h. The results are expressed as relative luciferase units compared with the cell transfected with β-catenin-FLAG vector with vehicle control. The relative firefly luciferase activity units (RLUs) were then measured and normalized with Renilla luciferase activity. Data are expressed as fold changes compared with pcDNA3.1-transfected cells. **c** The mRNA expressions of the direct target genes of Wnt/β-catenin including *c-Myc*, *Axin2*, *Cyclin D1*, and *Survivin*. Each value is the mean ± SEM; (*n* = 3). *P < 0.05, **P < 0.01, compared to the control group

The levels of protein and luciferase activity of β-catenin were assessed to examine whether AP increased PL-hMSC proliferation via the Wnt/β-catenin pathway. AP at 1 and 2.5 μM significantly increased the levels of active β-catenin proteins/total β-catenin at 24 h compared to the control (Fig. 3a). Of note, AP at 5 and 10 μM seemed to increase the expression of active β-catenin proteins, the results were, however, not statistically significant. Moreover, β-catenin activity levels were augmented by AP (Fig. 3b). The direct target genes of Wnt/β-catenin including *c-myc*, *Axin-2*, *Cyclin D1*, and *Survivin* were further examined. As shown in Fig. 3c, AP at 2.5 and 5 μM significantly enhanced the expressions of all these genes compared to the control whereas only *Cyclin D1* was increased by a lower concentration (1 μM). Based on these results, AP has proliferative effect on PL-hMSC, at least, via the Wnt/β-catenin pathway.

### AP promoted osteogenic differentiation

**Fig. 4:**
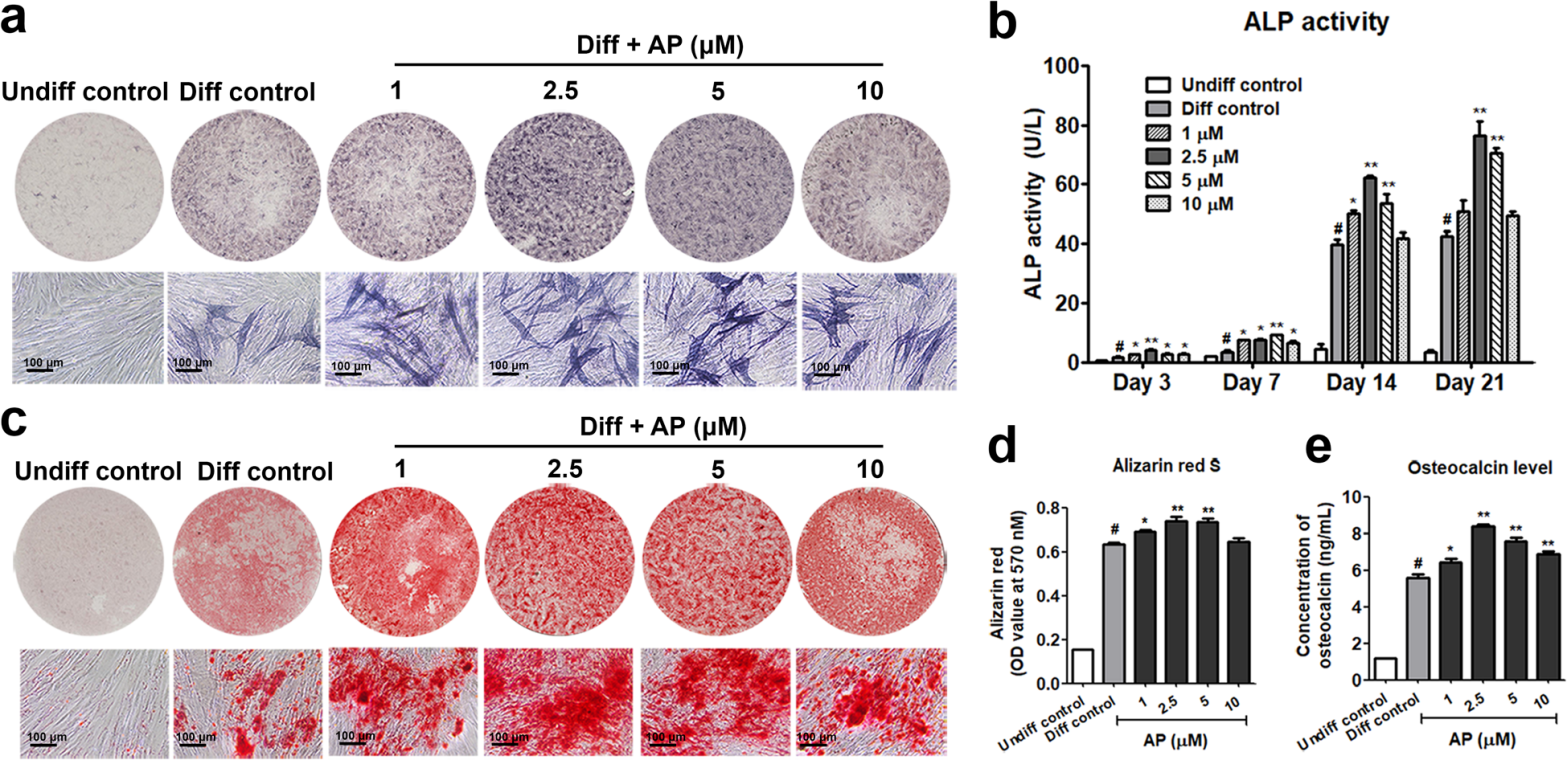
Effect of AP on osteogenesis of PL-hMSCs. **a** Alkaline phosphatase (ALP) expression in PL-hMSCs after culture in osteo-inductive conditions with or without AP (1–10 μM) for 14 days. **b** Quantitative intracellular ALP activity in PL-hMSCs after culture in osteo-inductive conditions with or without AP (1–10 μM) for 3-21 days. **c** Matrix mineralization visualized by alizarin red staining after culture in osteo-inductive conditions with or without AP (1–10 μM) for 21 days. **d** Quantification by released dye of the extracted Alizarin Red S stain was quantified by measuring absorbance at 570 nm. **e** Osteocalcin concentration in PL-hMSCs after culture in osteo-inductive conditions with or without AP (1–10 μM) for 21 days. Each value shown in **b**, **d**, and **e** represents the mean ± SEM (*n* = 3). #*P* < 0.05, the differentiation control group compared to the undifferentiation control group, *P < 0.05, **P < 0.01, the AP-treated group compared to the differentiation control group

To examine if AP promotes osteoblastic differentiation of PL-hMSCs, its effect on the expression and activity of alkaline phosphatase (ALP), a major marker enzyme in the early and middle stage of osteoblastic differentiation, was determined. After treatment with AP at the concentrations of 1, 2.5, 5, and 10 μM for 14 days, the expressions of ALP were evidently elevated compared to control (Fig. 4a). To confirm the osteogenic effect of AP, ALP activity assay was further quantitatively determined at various stages of differentiation. As anticipated, AP at concentrations of 1, 2.5, 5, and 10 μM showed increases in the expression and activity of ALP after treatment for 3, 7, 14, and 21 days. Consistent with the proliferative effect, 2.5 μM AP appeared to be most effective by day 14, i.e., ALP activity was increased by 57.14 ± 2.4%, compared with the control (Fig. 4b). In the late stages of osteoblast differentiation, the extracellular matrix was gradually mineralized due to calcium deposition or forming bone nodule. AP increased matrix mineralization of PL-hMSCs as evidenced by alizarin red staining and quantitative analysis (Fig. 4c, d). To compared as % changes with the osteogenic control, AP at concentrations of 1, 2.5, and 5 μM significantly increased calcium deposition by 9 ± 1.2%, 20 ± 1.0%, and 16 ± 2.4%, respectively. Moreover, bone formation was quantitatively determined by osteocalcin concentration assay. Of note, all doses of AP (1 to 10 μM) significantly increased osteocalcin concentration. Taken together, the results indicate that AP promotes both osteogenic differentiation and matrix mineralization of PL-hMSCs.

### Effects of AP on marker gene expressions of osteoblast differentiation and OPG/RANKL pathway

**Fig. 5:**
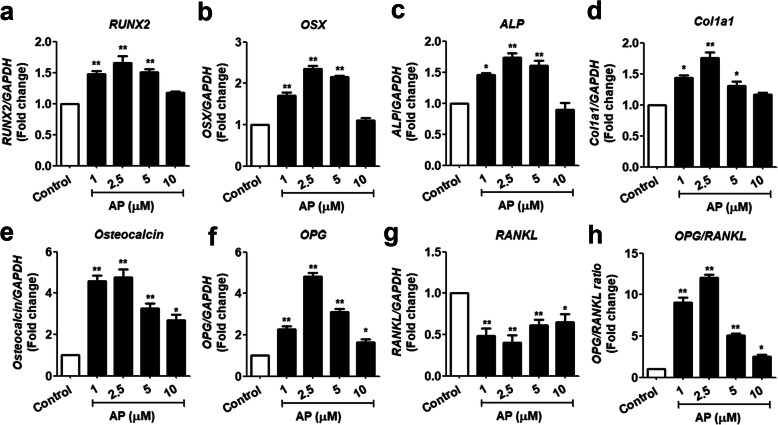
Effect of AP on osteoblast-marker gene expressions during osteoblast differentiation. The mRNA expressions of osteoblast marker genes in PL-hMSC-derived osteoblastic cells after culture in osteogenic media with or without AP at the indicated concentrations and times: Expressions of mRNA were determined relative to GAPDH by quantitative RT-PCR. **a** *RUNX2* (day 7). **b** OSX (day 7). **c** *ALP* (day 14). **d** *Col1a1* (day14). **e** *Osteocalcin* (day 21). **f** *OPG* (day 21). **g** *RANKL* (day 21). **h** The ratio of *RANKL/OPG* (day 21). Each value is the mean ± SEM; (*n* = 3). *P < 0.05, **P < 0.01, compared to the control group

On day 7 under the osteogenic condition, AP (1, 2.5 and 5 μM) caused a dose-dependent elevation of mRNA levels of *RUNX2* and *OSX*, which are the major transcription factors required at the early state for inducing osteogenesis in MSCs (Fig. 5a, b). The results suggests that AP affected an early phase of MSC differentiation into osteoblast lineage. In addition, the same dose range of AP increased the levels of the markers for middle phases of osteogenic differentiation, *ALP* (Fig. 5c). Furthermore, the late-stage markers, *Col1a1* and *Osteocalcin*, were significantly enhanced (*P* < 0.05) by days 14 and 21, respectively (Fig. 5d, e). These results clearly indicate that AP was involved in all stages of osteogenic differentiation of PL-hMSCs.

OPG and RANKL proteins secreted from osteoblasts are important molecules that regulate the balance between bone formation and bone resorption, and their mRNA expressions were further examined in osteoblasts derived from PL-hMSCs. As shown in Fig. 5f, g, AP at the concentrations of 1, 2.5, and 5 μM significantly increased mRNA levels of *OPG* compared to the control (p < 0.05) whereas the level of *RANKL* mRNA was suppressed by AP. The ratio of *OPG*/*RANKL* mRNA expression, consequently, was significantly increased (p < 0.05). The results suggest that the OPG/RANKL signaling pathway is involved in AP-induced osteogenesis of PL-hMSCs.

### Effect of AP on changes of gene expression during osteogenic differentiation

**Fig. 6:**
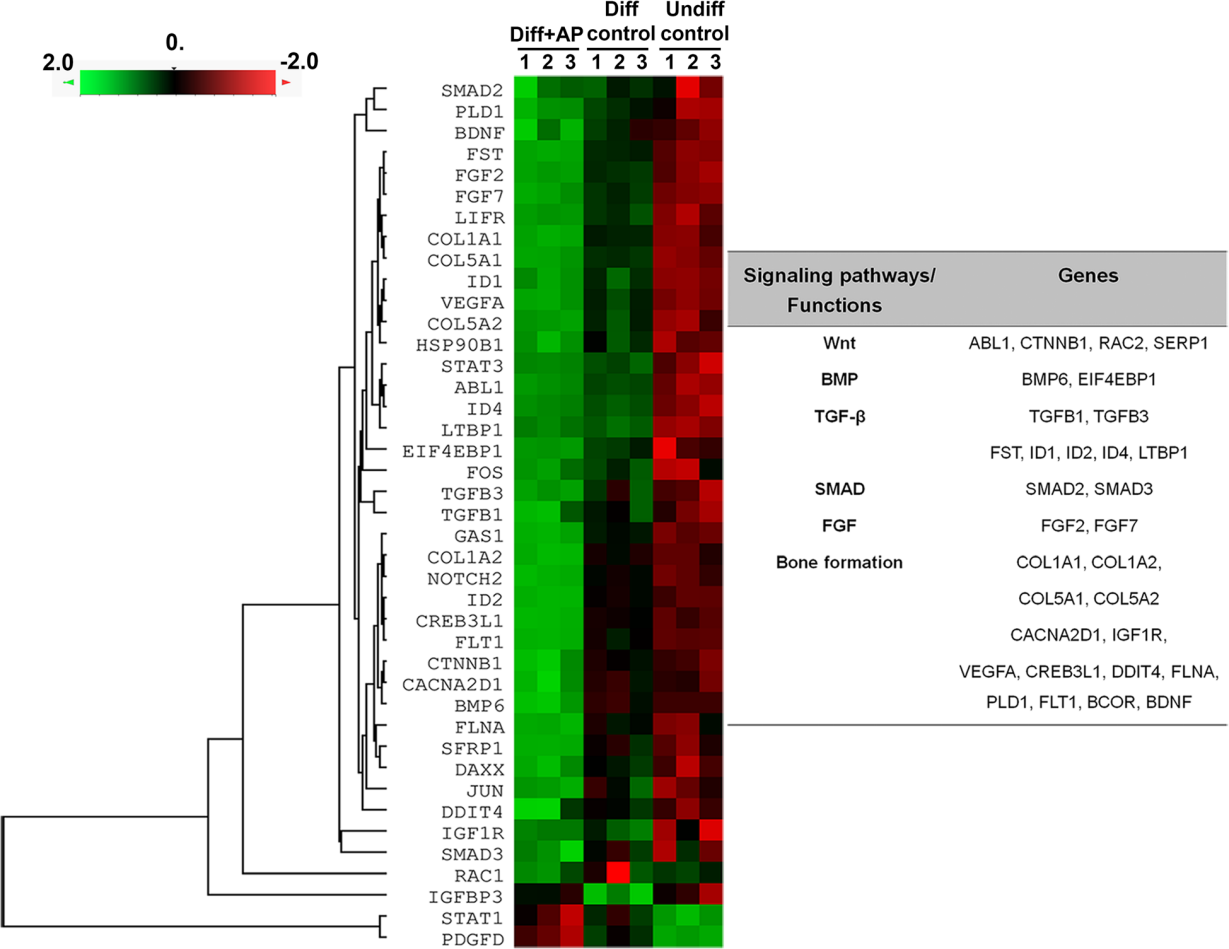
NanoString analysis of gene-related mRNAs during AP-enhanced osteogenic differentiation of PL-hMSCs. Triplicate samples of total mRNA isolated from PL-hMSCs at day 14 were analyzed using a NanoString Code Set. Left panel shows a heatmap of the 37 candidate genes differentially expressed between growth medium or undifferentiation control (Undiff control), osteo-inductive medium or differentiation control (Diff control), and osteo-inductive medium treated with 2.5 μM AP (Diff+AP). The right panel shows the summary of gene enrichment analysis of the upregulated genes by AP treatment that show statistical significance (*P* < 0.05) compared to differentiation control

In order to get into detail of the pathways that AP enhanced PL-hMSC differentiation into osteoblasts, we performed an analysis of digital gene expression with 770 genes screening using NanoString nCounter. Hierarchical clustering revealed significant changes in mRNA of 37 genes in AP-treated group compared to those of the control group (Fig. 6). These mRNA abundances were detected in ligands, receptors, or modulators of Wnt, TGFβ, SMAD, and BMP. Moreover, a set of genes related to bone formation were also significantly upregulated such as COL1A1, COL1A2, COL5A1, COL5A2, FGF2, FGF7, IGF1R, and VEGFA (Fig. 6 and supplement data 1). These findings suggest that the mechanisms of action of AP on osteogenic differentiation of PL-hMSCs are involved with several pathways and molecules that are important for osteogenesis.

## Discussion

Previously, AP has been shown to have pro-osteogenic effects on bone marrow stem cells of mice and rats, whereas it prevented TNFα-induced suppression of osteoblast formation and mineralization [32]. Also, we recently reported that AP enhanced the osteogenic capacity of mouse pre-osteoblast cell lines [30]. However, the effect of AP on the proliferation and osteogenic differentiation of PL-hMSCs are still unknown. This study shows, for the first time, that AP promotes the proliferation and differentiation of PL-hMSCs into bone cells via, at least, Wnt/β-catenin, TGFβ/BMP/SMAD, and FGF signaling pathways.

In recent years, human placental mesenchymal stem cells have become an attractive source of mesenchymal stem cells (MSCs) for tissue regeneration due to non-invasive procedures and less ethical criticisms [33]. In this study, the isolated PL-hMSCs met all the MSCs criteria set by the International Society of Cellular Therapy including adherence to plastic culture dishes, expression of a specific set of cell surface markers such as CD73, CD90, and CD105, but the absence of hematopoietic markers such as CD34 and CD45. In addition, they have the ability to differentiate in vitro into adipocytes and osteoblasts (Fig. 1) [13].

The key for using MSCs in therapeutic purposes is that they maintain the stemness of stem cells and can be expanded to meet the required amount of cells in laboratories. Although natural small molecules offer several compelling advantages, they might be unexpectedly cytotoxic to stem cells. Accordingly, a safe dose range was determined in this study. In the dose range of 1–10 μM, AP exhibited no cytotoxicity and showed the proliferative effect on PL-hMSCs. The results are consistent with previous report the effective dose of on proliferation less than 10 μM (4.46 and 8.92 μM) increased rat BM-MSC proliferation [32]. Although AP promote PL-hMSC proliferation, the specific MSC phenotype and surface protein markers were not affected suggesting that the stemness of the cells are maintained (Fig. 2c, d). Since Wnt signaling plays a critical role in adult proliferative and osteogenic differentiation of mesenchymal stem cells, we used Western blot and luciferase assay to determine the level and activity of β-catenin. As illustrated in Fig. 3, AP significantly augmented both the level and activity of β-catenin indicating that Wnt/β-catenin signaling plays a crucial role in this effect of AP. The results are consistent with our previous report in rat bone marrow stem cells [34]. Moreover, the mRNA expression levels of the direct target genes including *c-Myc*, *Axin2*, *Cyclin D1*, and *Survivin* were also elevated by AP (Fig. 3) confirming that AP exerted its proliferative effect at least via Wnt/β-catenin pathway.

The osteogenic differentiation of MSC is characterized by the expression of specific osteoblastic marker genes such as RUNX2, OSX, alkaline phosphatase (ALP), and type I collagen (Col1a1) followed by extracellular matrix synthesis and mineralization [35]. RUNX2, a master osteoblast transcription factor, is required to commit undifferentiated cells towards the osteoblast lineage and has a crucial role in regulating multiple genes involved in the osteogenesis [36]. In addition, osterix (OSX) is the second transcription factor required for osteoblast differentiation [37]. Thus, upregulations of both RUNX2 and OSX in this report suggest that AP induced changes of undifferentiated PL-hMSCs into the osteoblast lineage (Fig. 5). The results also corroborate the report that RUNX2 and OSX are downstream targets of Wnt/β-catenin signaling to promotes mesenchymal stem cells differentiate into immature osteoblasts [36]. Further, our results revealed the elevation of mRNA expression levels of both col1a1 and osteocalcin indicating that AP induced differentiation of the immature into mature osteoblasts. Also, AP promoted in vitro mineralization as indicated by alizarin red staining of mineralization nodules as well as the increase of osteocalcin production in PL-hMSCs (Fig. 4). Taken together, the increases in matrix mineralization and the bone specific genes and protein indicate the potential use of AP to promote PL-hMSC differentiation into mature osteoblasts and generate matrix mineralization.

The morphogenesis and remodeling of the bone depend on the integrated activity of osteoblasts that form bone and osteoclasts that resorb bone [38]. OPG produced by osteoblasts has a crucial role in preventing excessive activation of osteoclasts by RANKL, thereby maintaining bone homeostasis. Thus, the OPG/RANKL ratio may be used to indicate the potential of osteoblast in regulating bone remodeling process [39]. In our study, AP increased the OPG/RANKL ratio by increasing *OPG* expression level but decreasing *RANKL* expression suggesting that, in addition to promoting osteogenic differentiation, AP also enhanced the function of osteoblastic cells derived from PL-hMSCs.

Numerous signal transduction pathways and transcriptional factors, including the activation of Wnt/β-catenin, TGF-β/BMP, and SMAD signaling, have been extensively reported to regulate osteoblastic MSC differentiation [40, 41]. To determine the molecular mechanisms for the action of AP on PL-hMSC proliferation and differentiation, screening of genes involved in osteogenic differentiation was explored by a NanoString nCounter analysis. Results obtained from this data set revealed several importance pathways including Wnt, TGFβ, BMP, and SMAD. Moreover, other genes related to these pathways such as FGF2, FGF7, IGF1, IFGBP3, and VEGFA were also upregulated by AP. The results are, therefore, consistent with the role of Wnt signaling in the induction of Runx2-dependent transcription of TGFβ1 receptors [42, 43]. The central role of the TGFβ/BMP axis in regulating mesenchymal stem cell differentiation into the bone as well as extensive cross talk with other signaling pathways are well established [41, 44]. Of these, the activity of several genes including Hedgehog, FGF2, FGF7, and IGF1 appeared to be increased in parallel with TGFβ/BMP during cell differentiation. FGF2 regulates the expression of PC1, the primary enzymatic generator of pyrophosphate in mineralizing cells, by direct regulation of Runx2 suggesting that TGFβ/BMP and FGF2 signaling cooperate to promote matrix mineralization later in differentiation [41]. Surprisingly, this analysis showed the upregulation of FGF7, which has recently been reported to promote new bone formation in rats [45] and facilitate osteogenic differentiation of embryonic stem cells through activation of ERK/Runx2 signaling [46]. Moreover, the elevations of SMAD2 and SMAD3 in our study are in line with the notion that SMAD2 and SMAD3 are the common mediators for TGF-β signaling, which respond to TGF-β receptors in the process of coupling bone formation and bone resorption to maintain normal bone homeostasis [44]. In addition to the genes involved in cell proliferation and differentiation, the genes related to bone formation such as COL1A1, COL1A2, COL5A1, and COL5A2 were enhanced by AP suggesting a strong potential of AP in promoting differentiation of PL-hMSCs to bone required for bone regeneration.

Our results revealed that AP enhanced the β-catenin activity demonstrated by the increased level of non-phosphorylated β-catenin and the upregulation of several Wnt target genes. Moreover, AP also activated other signaling pathways including TGF-β, BMP, and FGF pathways, which have been known to play important roles during bone formation process. Taken together, we believe that the activation of these osteogenic promoting pathways might be responsible for the observed enhancement of osteogenic differentiation of PL-MSCs after AP treatment. However, it should be pointed out that our data from gene screening only gives clues for the possible molecules and pathways by which AP promoted proliferation and differentiation of PL-hMSCs. The detailed mechanisms require further investigations. Although the gestational tissue-derived MSCs, such as PL-MSCs could be easily obtained in large quantity using a non-invasive procedure, these MSCs generally have lower osteogenic differentiation capacity in comparison to BM-MSCs. We therefore believe that the AP could enhance the osteogenic differentiation of these MSCs, so the osteocyte derived from these MSC sources could be used for transplantation either as a single cell suspension or in combination with suitable matrices to treat patients with bone defects. However, additional in vivo studies are required to determine the efficacy of this approach.

## Conclusions

This report is the first to demonstrate that AP at the concentration of less than 10 μM promoted proliferation and osteogenic differentiation of PL-hMSCs via activation of, at least, Wnt/β-catenin signaling pathway. The findings provide strong evidence that AP is a novel effective agent for expansion and induction of the bone from PL-hMSCs for uses in stem cell-based therapy especially for bone regenerative medicine. Future studies are, however, required to investigate more specific mechanisms associated with the AP-mediated osteogenic differentiation of PL-hMSCs.

## Supplementary Information

**Additional file 1.** The summary of gene enrichment analysis of the upregulated genes by AP treatment that show statistical significance (P < 0.05) compared to differentiation control performed by NanoString analysis

## Data Availability

Data sharing not applicable to this article as no datasets were generated or analyzed during the current study.

## Abbreviations

AP: Andrographolide
ALP: Alkaline phosphatase
ARS: Alizarin Red S
BM-MSCs: Bone marrow-derived MSCs
BMP: Bone morphogenetic protein
BrdU: Bromodeoxyuridine
COL1A1: Collagen type I alpha 1 chain
COL1A2: Collagen type I alpha 2 chain
COL5A1: Collagen type V alpha 1 chain
COL5A2: Collagen type V alpha 2 chain
DMEM: Dulbecco’s modified Eagle’s medium
DMSO: Dimethyl sulfoxide
FBS: Fetal bovine serum
FGF: Fibroblast growth factor
FITC: Fluorescein isothiocyanate
GAPDH: Glyceraldehyde-3-phosphate dehydrogenase
HEK293T cell: Human embryonic kidney 293 cells
IC_50_: The half maximal inhibitory concentration
IGFBP3: Insulin-like growth factor-binding protein 3
IGF-1: Insulin-like growth factor 1
MSCs: Mesenchymal stem cells
MTT: 3-(4,5-Dimethylthiazol-2-yl)-2,5-diphenyl tetrazolium bromide
OPG: Osteoprotegerin
PBS: Phosphate-buffered saline
PE: Phycoerythrin
PL-hMSCs: Human placenta-derived mesenchymal stem cells
RANKL: Receptor activator of nuclear factor kappa-Β ligand
RUNX2: Runt-related transcription factor 2
TGF-β: Transforming growth factor-beta
VEGFA: Vascular endothelial growth factor A
